# Retraction: Kweichow Moutai ameliorates alcohol‐induced liver fibrosis in mice by targeting the NFκB pathway

**DOI:** 10.1002/fsn3.3720

**Published:** 2023-10-31

## Abstract

Yang, T, Feng, Y, Zhang, Y, et al. Kweichow Moutai ameliorates alcohol‐induced liver fibrosis in mice by targeting the NFκB pathway. *Food Sci Nutr*. 2020; 8: 4214–4222. https://doi.org/10.1002/fsn3.1716.

The above article, published online on June 15, 2020 in Wiley Online Library (wileyonlinelibrary.com), has been retracted by agreement between the journal Editor in Chief Y. Martin Lo, and Wiley Periodicals LLC. The retraction has been agreed because of concerns that the figures were duplicated, affecting the interpretation of the data and results presented.

Yang, T, Feng, Y, Zhang, Y, et al. Kweichow Moutai ameliorates alcohol‐induced liver fibrosis in mice by targeting the NFκB pathway. *Food Sci Nutr*. 2020; 8: 4214–4222. https://doi.org/10.1002/fsn3.1716.

The above article, published online on June 15, 2020 in Wiley Online Library (wileyonlinelibrary.com), has been retracted by agreement between the journal Editor in Chief Y. Martin Lo, and Wiley Periodicals LLC. The retraction has been agreed because of concerns that the figures were duplicated, affecting the interpretation of the data and results presented.

